# Content accuracy and readability of dietary advice available on webpages: A systematic review of the evidence

**DOI:** 10.1111/jhn.13395

**Published:** 2024-11-26

**Authors:** Evaggelia Fappa, Mary Micheli

**Affiliations:** ^1^ Department of Nutrition and Dietetics, School of Health Sciences University of the Peloponnese Kalamata Greece

**Keywords:** diet, information, nutrition, quality, readability, systematic review

## Abstract

**Background:**

Concerns have been raised regarding the quality of health information published on the World Wide Web, while studies accumulating similar evidence for nutrition‐related information are scarce. The present review aimed to systematically accumulate and discuss the findings of studies evaluating the content quality of websites publishing nutrition‐related information, based on the PRISMA statement.

**Methods:**

Studies that have assessed the accuracy and readability of dietary advice published on websites/webpages were evaluated. The SPIDER framework was used for the systematic search of studies, and those that evaluated websites/webpages that did not contain nutritional information, were videos or social media, referred to health professionals, or contained information on a specific theme (e.g., dietary supplements), were excluded.

**Results:**

Twenty‐nine studies were included in this review, assessing information, published mainly in English, on 18 different diet‐related topics. Twenty of them classified websites and reported, also, results per category. Inconsistent information has been found on 16%–49.6% of websites, with this percentage rising to 54%–94% in the case of ‘weight loss’ information. Purely congruent with guidelines information was found on 18%–39.7% of websites. Commercial sites were inferior in terms of quality to the rest. The readability level was estimated as higher than the recommended in 9 out of 11 studies that assessed it.

**Conclusions:**

Results of the present systematic review indicate that inaccurate and hard‐to‐read dietary advice is found on many websites, regardless of the dietary topic.

## Key points

- Inconsistent information has been found on 16%–49.6% of websites, while in the case of ‘weight loss’ this percentage rose to 54%–94%.
- Purely congruent with guidelines information was found on 18%–39.7% of websites.
- Difficult‐to‐comprehend information was found on most of the websites based on readability levels.

## Graphical abstract

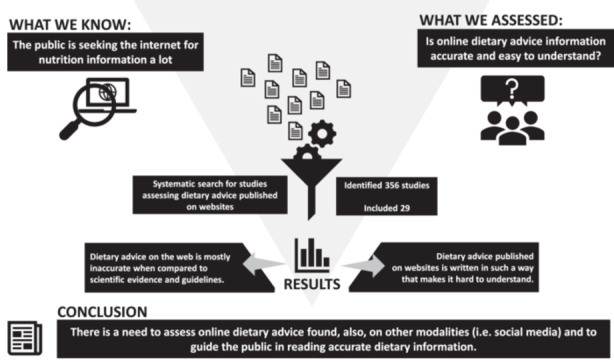

## INTRODUCTION

European data show that 53% of European citizens aged 16–74 years search the Internet for health information,^1^ with diet and nutrition being among the most popular ones.^2^, ^3^ And it has been found that approximately 70% of people who seek nutrition‐related information online believe that this information would help them follow a healthier diet.^4^ Furthermore, half of those in the online search of dietary information stated that they wanted to change their diet and physical activity based on that information.^5^ Also, seeking nutritional information on the Internet has been linked to disordered eating, in adolescents.^6^ At the same time, most of the people who seek health‐ and nutrition‐related information on non‐institutional websites lack the ability to assess the quality of the nutrition information they retrieve, and only 16.0% of them discuss the information they find with a healthcare professional.^5^ Some researchers believe that dietary knowledge is considered a prerequisite for a person to improve his/her dietary habits, even though this might not be sufficient to achieve behavioural change.^7^ Thus, conflicting information constitutes a barrier to improving nutrition.^8^ Considering, the increasing trends in the prevalence of diet‐related chronic diseases^9^ showing that people are experiencing serious difficulties in adhering to dietary guidelines,^10^ efforts should be made to support scientifically the communication of accurate dietary information to the public.

With the internet being a large uncontrolled repository of health information contributing to the increase of the seeker's knowledge^11^ numerous studies have assessed the quality of online health information. Consequenlty, several systematic reviews have gathered that information to provide an overall result on the quality of online information, to explore online health information‐seeking behaviour, or to investigate the effect of this information on decision making.^12^, ^13^, ^14^ The same does not apply to dietary information, even though various studies have assessed relevant content published online.^15^, ^16^, ^17^, ^18^, ^19^ To our knowledge, only one systematic review has been published so far that has accumulated and presented the quality and accuracy level of nutrition‐related information found in online environments.^20^ Quality, defined as the reliability of information, was evaluated based on criteria such as citing references, while accuracy was captured as the congruence of online content with evidence‐based guidelines. Researchers found that the quality and accuracy of online nutrition‐related information were similar on social media and websites and assessed as low by half of the studies included in their review. They also found great inconsistency between studies' findings regarding the quality and accuracy of information based on the publisher (government, commercial, academic, etc.). This review included studies assessing various types of nutritional information (i.e., healthy eating, dietary patterns, nutrients, nutritional requirements, nutritional composition of foods, nutritional supplements, health outcomes associated with foods and dietary patterns, food safety, food ethics and cooking) found both on websites and social media.

Nowadays, various modalities of communicating nutritional information have been added to the traditional ones, such as television, websites, social media and podcasts.^21^ However, studies show that the majority of people search, still, for dietary information on websites compared to social media and that there are differences in demographic characteristics of searchers based on the modality they refer to for this kind of information.^22^, ^23^ In specific, people of higher education levels, are more likely to refer to websites and newspapers than to videos and television. At the same time, the readability level of online health information has proven inappropriate for the public.^24^ To our knowledge, there is no previous review gathering the relevant data for dietary information found on websites. Thus, the aim of the present systematic review was to accumulate and discuss the findings of all research studies presenting data on the content accuracy, and readability of online information found on webpages that provide advice on diet. A second aim was to explore if there were any differences between the various types of websites in content accuracy.

## MATERIALS AND METHODS

To ensure methodological thoroughness of the review process, the Preferred Reporting Items for Systematic Reviews and Meta‐Analyses (PRISMA) 2020 statement checklist and flowchart^25^ were used.

To systematically approach the search strategy, we chose to use the Sample, Phenomenon of Interest, Design, Evaluation, Research type (SPIDER) framework^26^ over the PICOS framework, which is the most commonly used in quantitative health sciences research. This decision was based on the fact that the purpose of the current study was not to assess intervention or exposure in human subjects.^27^ Table 1 outlines the key components of our research question of ‘How is the quality of information on webpages with nutritional content being evaluated?’ using the SPIDER framework.

**Table 1:** SPIDER search strategy tool implementation.

Parameter	Content
Sample	Websites/Webpages with consumer‐oriented nutritional content
Phenomenon of interest	Quality of information
Design	Descriptive, cross‐sectional
Evaluation	Scientific agreement, formality of content
Research type	Quantitative

### Inclusion and exclusion criteria

In this systematic review, we included cross‐sectional studies published in English, that evaluated websites'/webpages' quality of information of dietary advice addressed to the public. Studies assessing the quality of information with quantitative methods, using designated validated instruments or appropriate checklists that evaluated content against formal guidelines or evidence‐based knowledge and practices were included. No time limit was applied for the literature search. The SPIDER tool laid the foundation for the inclusion of eligible studies. Studies were excluded if they: (a) did not assess nutritional recommendations (i.e., reporting results on dietary supplements, dietary fat intake, herbs and natural remedies, physical activity or medical treatments); (b) presented information addressed to health professionals (i.e., websites of scientific journals); (c) assessed the quality of information based on public's criteria; (d) included only certain types of sources (i.e., assessing information presented only on printed media).^12^

### Study selection strategy

Both authors conducted independently, a comprehensive search for studies with a last check on 11th March 2024, in some of the most used academic research databases available, namely PubMed; Ovid (Embase); Science Direct and Google Scholar. For Ovid and Google Scholar searches, the authors conferred and agreed to review several SERPs (Search Engine Result Pages) that were producing relevant results. As both Ovid and Google Scholar rank their results based on relevancy, we expected that this strategy was not counter‐productive to the purpose of our study. As such, when three consecutive SERPs did not contain any relevant papers—on the quality of nutrition information on the web—the authors ended the search.

Based on the chosen search strategy framework, the keywords encompassed five (5) thematics (concepts):

- **S** ‐ (1) (nutrition* OR food OR diet*) AND (2) (Internet OR website * OR online)
- **PI** (3) (quality OR reliability OR accuracy) AND (4) (information OR advice OR guide*)
- **E** ‐ (1) (evaluation OR assessment OR analysis)

In PubMed, Science Direct and Ovid, we focused on concepts (1) and (4) being in the title section, as we wanted to narrow down the number of results, while the rest anywhere possible, while in Google Scholar, we did not use any specific filters and relied solely on logical syntax.

Mendeley software was used to manage references. The articles were screened for eligibility in two stages: first, by assessing relevance based on the study's title, and second, if the study passed the first screening by reading the abstract. Any uncertainties regarding eligibility were resolved through discussion.

### Screening

Search results were acquired as Comma Separated Values files (.csv) wherever possible, or otherwise downloaded and saved in MS word files. Both sources were then imported into an MS Excel file. One of the authors (M. M.) manually screened the results for duplicates. After the removal of duplicates, both authors (E. F. and M. M.) independently assessed the eligibility of the studies, based on title and abstract content. Any ambiguities were discussed after the completion of the independent screenings, and a consensus was reached. Both authors screened eligible studies based on full‐text content to meet the inclusion criteria. No studies were excluded at this point.

### Data extraction

For the retrieval of information relevant to the aims of this systematic review, one author (M. M.) extracted necessary data from an MS Excel workbook. Fourteen items were extracted in total from each study, as shown in the summary in Table 2. After the initial extraction process, the second author (E. F.) reviewed the extracted data, and any inconsistencies or missing information were reviewed by both authors and incorporated appropriately.

**Table 2:** Data extracted from the research papers included in the review.

Category	Item
Basic information	– Author(s) of publication – Year of publication – Topic of nutritional recommendations – Number of assessed sites – Number of evaluators
Methods of evaluation	– Search engines used by researchers (if applicable) – Web browsers used by researchers (if applicable) – Search terms entered in search engines – Criteria for content quality of dietary recommendations – Criteria or instruments for assessment of information readability level – Classification categories for websites (government, organisation, commercial, etc.) (if applicable)
Study results	– Key findings on content accuracy – Key findings on content accuracy based on website classification – Key findings on readability

## RESULTS

### Studies included

**Figure 1:**
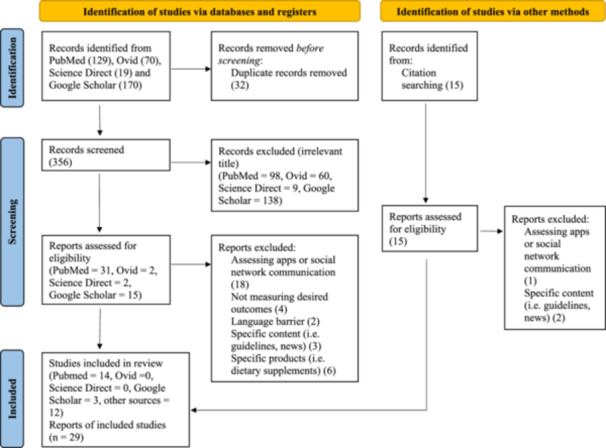
PRISMA flowchart.

The process of screening and selecting the studies is summarised in Figure 1. The literature search yielded 129 studies via PubMed, 70 studies via Ovid, 19 studies via Science Direct and 170 studies via Google Scholar, which arrived at 356 records after removing duplicates. We screened for title relevance and selected 50 potentially eligible studies, of which 33 were excluded due to not meeting our inclusion and exclusion criteria. Twelve additional studies were identified through the reference lists of initially retrieved articles and were added to the assessment pool. Twenty‐nine studies were included in this systematic review.

### Studies characteristics

All 29 studies had a cross‐sectional design, assessing the nutritional information content of webpages using relevant tools or scales. Concerning the specific content assessed, information on 18 diet‐related topics was evaluated in all studies (Table 3). The topics mostly evaluated were the ‘dietary guidelines for healthy eating’ and ‘dietary guidelines for pregnancy’. It should also be noted that, except for eight studies, all assessed webpages were written in English (Table 3). The evaluation methodology of the studies consisted of two parts, each of which comprised specific steps. The first part identified the websites to be evaluated and consisted of the following steps: (1) generation of search terms, (2) search engine and browser selection and (3) webpage selection.

**Table 3:** Methodological characteristics of the studies included in the systematic review.

Characteristics	*n*	%	References
Nutrition‐related topic assessed			
Cancer	3	10.3	[18, 28, 29]
Coeliac disease	1	3.4	[30]
COVID‐19	1	3.4	[16]
Dementia	1	3.4	[17]
Health and disease (various subjects)	1	3.4	[31, 32]
Healthy diet for the general public	6	20.7	[33, 34, 35, 36, 37]
Irritable bowel syndrome	1	3.4	[38]
Kidney stones	1	3.4	[39]
Multiple sclerosis	1	3.4	[40]
Osteoporosis	1	3.4	[41]
Polycystic Ovary Syndrome	1	3.4	[42]
Pregnancy	3	10.3	[43, 44, 45]
Renal disease	1	3.4	[46]
Type 2 diabetes mellitus	2	6.9	[47, 48]
Veganism	1	3.4	[49]
Weight loss	4	13.8	[15, 19, 50, 51]
Eating disorders	1	3.4	[52]
Language of website content			
English	15	51.7	[17, 18, 28, 30, 34, 35, 37, 38, 40, 41, 42, 43, 45, 46, 49]
Assumed English	7	24.1	[15, 31, 36, 39, 44, 47, 48]
French	1	3.4	[35]
German	2	6.9	[19, 29]
Italian	1	3.4	[16]
Spanish	1	3.4	[50]
Persian	1	3.4	[32]
Not specified	2	6.9	[33, 51]
Browser used to search for websites			
Chrome	5	17.2	[15, 17, 18, 40, 50]
Netscape Navigator version 2.0	1	3.4	[33]
Various (Internet Explorer, Safari, Mozilla Firefox, Chrome)	1	3.4	[42]
Not specified	22	75.9	[16, 19, 28, 29, 30, 31, 32, 34, 35, 36, 37, 38, 39, 41, 43, 44, 45, 46, 47, 48, 49, 51]
Search engines used to retrieve websites			
Google	12	41.4	[15, 16, 17, 18, 19, 28, 29, 35, 39, 45, 50, 51]
Google and Yahoo	2	6.9	[31, 48]
Google, Yahoo and Bing (or MSN)	9	31.0	[30, 37, 40, 42, 43, 44, 46, 47, 49]
Various	4	13.8	[34, 36, 38, 41]
Unspecified	2	6.9	[32, 33]

Abbreviations: COVID‐19, Coronavirus disease 2019; MSN, Microsoft network.

#### Generation of search terms

In most studies (*n* = 22), keywords were chosen by the researchers, either based on the assumption that these are most likely to be used by the public,^33^ that these will capture a broad range of available information,^31^, ^34^ that these are the top trending terms in Google Trends on the topic,^40^, ^43^ or without providing any rationale for the choice. In three studies,^15^, ^19^, ^50^ the researchers consulted a group of individuals to produce keywords relevant to the study's topic, complemented by Google Autocomplete and Google Trends services to generate a set of queries. Lambert et al.^46^ and Keaver et al.^18^ also consulted a group of patients to decide the search terms in their study, whereas Google Trends was used by Storr et al.^43^ to select the most popular search terms for their study topic. Finally, on one occasion, the Search Intent Modelling report was used to identify search phrases used for a topic.^45^

#### Search engine and browser selection

Of the 29 studies, only seven mentioned the browser used, five of which were chrome browsers, since it was the most commonly used browser,^15^, ^17^, ^18^, ^40^, ^50^ and Netscape Navigator^33^ (Table 3). Regarding the search engines used by the researchers to retrieve the websites, Google search was used solely in 12 studies^15^, ^16^, ^17^, ^18^, ^19^, ^28^, ^29^, ^35^, ^39^, ^45^, ^50^, ^51^ and in combination with other engines in 16 cases (Table 3). In all but one study, the popularity of the search engine/s was the selection criterion. Only Alfaro‐Cruz et al.^38^ used the DuckDuckGo and Ixquick engines because they were non‐tracking. MSN, Ask and Bing were selected by the investigators in addition to Google for both the high visiting rate and because each search engine uses a different algorithm to retrieve and rank websites. The majority of the researchers based their choice of search engine(s) and browser on what was most used by the public.

#### Website selection

Using the queries developed in Step 1 and the search engines selected in Step 2, a search for the relevant websites was conducted for all studies. Then, a selection of websites most likely to be visited by users was made to end up with a sub‐group of websites to be evaluated. To achieve this, researchers included several websites retrieved as the first result in each search engine for each search term or those appearing on a number of pages of the search engine. In one study, the authors included websites with the most traffic for health and nutritional information^35^ without explaining how this was achieved. Seven studies used the first pages that appeared as a result of the search, with the majority having included the results of the first two pages of the search engine for each term.^40^, ^42^, ^43^, ^44^, ^45^, ^46^, ^51^ Fifteen studies used a number of first results from each search engine, ranging from 3 to 100, with the most common method being to include the top 30 websites from each search engine and each search term or the top 100.^15^, ^16^, ^17^, ^18^, ^19^, ^29^, ^31^, ^36^, ^37^, ^38^, ^41^, ^47^, ^48^, ^49^, ^50^

#### Exclusion criteria

Except for one study,^32^ researchers applied one or more exclusion criteria to reach the final group of websites to be evaluated. Only one study did not exclude any website because there were a limited number of websites on the topic.^32^ Four studies used only one exclusion criterion,^34^, ^39^, ^42^, ^51^ one used two,^31^ and the remaining three or more criteria. The most frequently used criterion was duplication of the website applied in 16 studies,^21^, ^22^, ^25^, ^26^, ^27^, ^28^, ^31^, ^32^, ^34^, ^36^, ^40^, ^42^, ^45^, ^47^, ^48^, ^49^ followed by having a non‐functional link/invalid address,^15^, ^17^, ^18^, ^31^, ^33^, ^36^, ^37^, ^40^, ^41^, ^43^, ^49^, ^50^ restricted access due to the need for registration or payment,^16^, ^17^, ^18^, ^29^, ^36^, ^37^, ^46^, ^48^, ^49^, ^51^ irrelevant content,^16^, ^18^, ^28^, ^29^, ^31^, ^44^, ^46^, ^49^, ^50^ aiming to sell a specific product,^15^, ^29^, ^35^, ^36^, ^37^, ^45^, ^48^, ^50^ meant for professionals such as scientific papers in medical journals,^17^, ^18^, ^30^, ^36^, ^37^, ^40^, ^44^, ^48^ or being linked to other sites.^36^, ^37^, ^48^ Other exclusion criteria consisted of being a list of links, URLs to videos or forums, partially covering the topic, being in another language, and so forth.

#### Website sample and classification

As a result, 8 to 693 webpages were evaluated by research groups in the 29 studies. There was great variation in the number of websites assessed that did not appear to be related to the topic of interest.

Then, before proceeding to evaluation, except in four cases,^38^, ^44^, ^47^, ^48^ research groups classified the websites to be assessed. The researchers used various numbers and types of categories for classification. Regarding the number of classification categories, which ranged from 2 (private or organisational)^32^ to 10 (academic, dietitians, government bodies, medical organisations, media outlets, commercial, patient support organisations, medical doctors, naturopaths and others),^46^ with the rest, falling into 3 to 7.^15^, ^16^, ^17^, ^28^, ^30^, ^31^, ^33^, ^34^, ^35^, ^36^, ^37^, ^39^, ^40^, ^42^, ^43^, ^45^, ^49^, ^50^ Regarding the categories used, in 17 cases, websites were classified primarily as commercial and others, with the category ‘others’ consisting of various sub‐categories such as institutional, medical, pharmaceutical, governmental or news. Some of those categories (i.e., organisational, professional, governmental and private/personal) were also used by the rest of the studies.

#### Website evaluation

Following the above, an evaluation of webpages was conducted with various tools and methods (Table 4). The content of websites was assessed mainly by comparing it to evidence‐based guidelines such as the Guide to Dietary Approaches to Stop Hypertension (DASH),^52^ Dietary Guidelines for Americans,^53^, ^54^, ^55^ the Joint Position Statement of the American Diabetes Association (ADA), the American Association of Diabetes Educators and the Academy of Nutrition and Dietetics,^56^ with a self‐made scale (Table 4). This kind of assessment was performed by allocating a mark based on whether the information was complete and/or accurate compared to the evidence‐based guidelines. Other ways used to assess the quality of the information published was using a generic tool to evaluate various aspects of the websites, including content,^17^, ^18^, ^31^, ^38^, ^41^, ^49^ or metadata, such as date of last update, citation of supporting literature, editorial team, and so forth.^32^, ^40^, ^51^ The tool used most^17^, ^29^, ^31^, ^36^, ^38^, ^41^, ^46^, ^49^ to assess quality content was DISCERN,^57^ either alone or in combination with self‐made scales. This tool consists of 16 questions scored on a five‐point Likert scale. An overall DISCERN quality rating score of ‘2’ or below indicates poor quality; a rating of ‘3’ indicates fair quality; and a rating of ‘4’ or above indicates the material has minimal shortcomings and is of good quality. Other methods used for the same goal were the EQIP^58^ and the IPDAS tool^40^ Regardless of the assessment method, in most of the studies, two independent assessors scored the content, and the mean outcome was used, and when a discrepancy occurred, a third person contributed.^51^ In five cases three assessors were used^16^, ^38^, ^43^, ^47^, ^50^ and in six studies this information was not provided.^28^, ^30^, ^32^, ^39^, ^45^, ^48^ The background of assessors, when specified, was either health‐related or technical.^31^, ^41^, ^42^

**Table 4:** Studies' accuracy of information and readability tools used.

Methods	*n*	%	References
Information quality measurement			
DISCERN	7	24.1	[17, 31, 36, 38, 41, 46, 49]
EQIP	1	3.4	[31]
IDPAS	1	3.4	[18]
Self‐constructed questionnaire	18	62.1	[15, 16, 19, 28, 29, 30, 32, 33, 34, 37, 40, 42, 43, 44, 47, 48, 50, 51]
Qualitative assessment	3	10.3	[35, 39, 45]
Readability evaluation			
FRES	8	27.6	[18, 19, 28, 34, 36, 43, 45, 46, 49]
F‐KGLS	8	27.6	[18, 34, 36, 38, 43, 46, 47, 49]
Online tool	1	3.4	[15]
GFI	2	6.9	[43, 46]
SMOG	2	6.9	[43, 46]
Coleman–Liau	1	3.4	[46]
Automated Readability Index	1	3.4	[46]
Linsear Write Formula	1	3.4	[46]
Gulpease Readability Index	1	3.4	[16]
Direct word count	1	3.4	[16]
Not performed	16	55.2	[17, 29, 30, 31, 32, 33, 35, 36, 39, 40, 41, 42, 44, 48, 50, 51]

Abbreviations: EQIP, Ensuring Quality Information for Patients; FRES, Flesch Reading Ease Score; F‐KGLS, Flesch‐Kincaid Grade Level Score; GFI, Gunning Fog Index; IPDAS, International Patient Decision Aids Standards; SMOG, Simple Measure of Gobbledygook.

Thirteen of the 29 studies assessed, also, readability,^15^, ^16^, ^18^, ^19^, ^28^, ^34^, ^36^, ^38^, ^43^, ^45^, ^46^, ^47^, ^49^ using one or more tools (Table 4). Eight studies used the Flesch‐Kincaid Grade Level (F‐KGLS)^59^ alone or in combination with other tools.^18^, ^34^, ^36^, ^38^, ^43^, ^46^, ^47^, ^49^ The F‐KGLS index corresponds to the grade level of the educational system in the USA (scoring 0–18). A 6–8 score is considered a standard for most studies as the National Institutes of Health recommends that the readability of health education material be no higher than 6th to 8th grade levels.^60^ Similarly, either alone or in combination, the Flesch Reading Ease Score (FRES)^61^ was used in eight studies.^19^, ^28^, ^34^, ^36^, ^43^, ^45^, ^46^, ^49^ A high (up to 100) FRES score indicates that the material is easier to understand, whereas a lower score indicates that the text is more difficult to read. A score of 60–70 is considered ‘plain English’ and ‘easily understood by 13‐ to 15‐year‐olds’. The Gunning Fog Index formula,^62^ SMOG Index^63^ and http://www.readable.com were used in two studies. Other tools used were the Coleman–Liau Index,^64^ Automated Readability Index,^65^ Linsear Write Formula^66^ and Gulpease Readability Index.^67^ In all these tools, a higher score indicates higher readability and understandability.

### Results on content accuracy

Self‐made scales have been used in most studies to assess content quality. Outcomes varied, with some of the studies presenting results based on scores and others on qualitative data, while variation also exists in the scoring ranges (Table 5). Regardless of the above, inconsistent information has been found on 16%–45.5% of websites on healthy nutrition.^33^, ^43^, ^45^ This percentage rose to 54%–94% in studies assessing websites publishing information on weight loss.^15^, ^50^ In contrast, purely congruent, with guidelines, information was found on 18%–39.7% of websites.^15^, ^35^, ^43^, ^45^ Mixed (accurate and inaccurate) advice was also found in 22.8%–64% of sites.^30^, ^35^, ^43^, ^45^ When a content quality score was calculated, most studies reported values below half and closer to one‐third of the maximum mark^15^, ^47^, ^50^ or low percentages of sites achieving an acceptable score.^32^ Regarding the number of recommendations mentioned on websites, only on a few occasions, they were fully and correctly presented.^28^, ^37^, ^39^, ^44^, ^48^ In two studies with cancer prevention as the topic of interest, only 10.3% of the sample sites included all 10 recommendations, 28.9% included 5–10 of them and a mean of 7 out of the 10 were present in the sites mentioning nutrition guidelines.^18^, ^28^ In another study, the mean matching score of websites to recommendations ranged from 33.5% to 47.2%.^37^

**Table 5:** Results of studies on quality and readability.

References	Topic of interest	Results on quality	Results on readability
Gholizadeh et al.^32^	Nutrition and diet therapy	66.7% of websites had mediocre content quality score and 33.3% acceptable score.	Not assessed
Davison Karen^33^	Healthy diet for the general public	45.8% of assessed websites provided specific dietary recommendations.	Not assessed
		45.5% of assessed websites provided information inconsistent with Canadian standards.	
Sutherland et al.^34^	Healthy diet for the general public	Content accuracy was higher for sites retrieved from the US Department of Health and Human Services Web portal (Focused Search – FS) versus those found in Google, Lycos & Alta Vista (General Search – GS) (mean scores: 2.63/3 vs. 1.84/3, *p* < 0.0001)	*F‐KGLS* was moderate (mean scores: 10.2 vs. 12.1, not significant). *FRES* was similar in GS and FS (mean scores: 54.8% vs. 37%, not significant). Over 75% of all websites were at least fairly difficult to read (score <50), while none were easy to read (score >80).
Ostry et al.^35^	Healthy diet for the general public	31.0% of websites were congruent with guidelines, 10.3% were incongruent, 24.8% contained mixed advice, and 33.9% had no nutritional information relevant to the guidelines.	
Hirasawa et al.^37^	Healthy diet for the general public	The overall quality of information scores ranged between 33.5% and 47.2% for all websites.	
Hirasawa et al.^36^	Healthy diet for the general public	The mean DISCERN score was 33.8/80 (‘poor’). Νο site was rated ‘excellent’ (score between 63 and 80) and one was rated ‘good’ (score between 51 and 62).	*FRES* had a mean of 55.9% (‘fairly difficult’) for all websites. A 37.5% of all websites scored 60%–70% (‘standard’, recommended). *F‐KGLS* had a mean of 7.2 for all websites (within the 6 to 8 grade recommended).
Storr et al.^43^	Pregnancy	39.7% of webpages contained accurate advice, 37.5% contained inaccurate advice and 22.8% contained a mix of accurate and inaccurate advice.	Mean Grade Level calculated by *F‐KGLS, GFI and SMOG* was 11.8 ± 2.48, with just 0.5% of webpages at or below the recommended reading level of grade 6. *FRES* mean was 51.6 ± 12.38 (‘fairly difficult’), with 6.6% of webpages scoring >70.
Cannon et al.^44^	Pregnancy	Mean score for websites containing nutritional information was 8 out of 16 dietary recommendations for pregnant women.	
Sidnell and Nestel^45^	Pregnancy	Overall accuracy score was 83%. 64% of webpages contained a mix of accurate and inaccurate advice, 18% were complete and accurate, 16% were entirely inaccurate and 2% lacked any relevant advice.	*FRES* median was 55%, while 33% of the webpages scored >60%.
		**Specific topics accuracy:** *On foods to avoid during pregnancy*, 45% were accurate, 46% were missing information and 9% were inaccurate. *For foods to eat during pregnancy,* 43% were accurate, 42% were missing information and 15% were inaccurate. As for *supplements during pregnancy*, 50% were accurate, 21% were missing information and 29% were inaccurate. Pairwise differences in the percentage accuracy by dietary theme were not statistically significant.	**Specific topics readability:** FRES > 60% was achieved for 60% of webpages on *what to avoid*, FRES < 60% (‘fairly difficult’) was attained by 61% of webpages on *what to eat*, and 81% on *supplements*. Pairwise differences in readability by dietary theme were found; pages on foods to avoid were easier to read than pages on supplements (*p* < 0.001), and pages on foods to eat were also easier to read than those on supplements (*p* < 0.001).
El Jassar et al.^49^	Veganism	The mean overall quality score of information was 41.6/80.0 (fair). 13.4% of websites scored excellent, 9.0% good, 31.3% fair, 32.8% poor and 13.4% very poor.	*FRES* mean was 63.3 ± 9.6 (close to standards), although 56.7% of websites had a score <65 (recommendation). *F‐KGLS* mean was 6.6 ± 1.7, while 13.4% had a score >8 (more difficult than recommended).
England and Nicholls^30^	Coeliac disease	The mean score of content accuracy regarding a gluten‐free diet was 6.7 ± 3.4/14, 44.4% of websites scored >7/14, 11.1% >10/14, and 19.5% <4/14, 3.2% of sites provided wrong information on where gluten is found.	Not assessed
Bernard et al.^47^	Type 2 diabetes mellitus	The mean score of information overall quality was 11.4 ± 7.75/30.	*F‐KGLS* was 11.5 for ‘medical nutrition therapy for diabetes’, 6.43 for ‘how to eat with diabetes’ and 6.29 for ‘diabetes diet’.
		**Specific search term evaluation:** score of 10.0/30 for ‘how to eat with diabetes’, 10.7/30 ‘for diabetes diet’, and 13.0/30 for 'medical nutrition therapy for diabetes’ (non‐statistically significant differences).	
Post and Mainous^48^	Type 2 diabetes mellitus	The mean content quality score was 3.56 ± 2.2/11 and did not differ for up‐to‐date sites versus non‐up‐to‐date websites.	Not assessed
Joshi et al.^41^	Osteoporosis	Average (2 raters) content scores ranged from 2.01 to 2.91/5 based on the type of website (i.e., .org, .gov, .com)	Not assessed
Gkouskou et al.^31^	Health and disease (nutrition and dysphagia and children, herbs and common cold, Mediterranean diet, sports nutrition)	Web site credibility (accuracy, scope, source, relevance and currency of information) ranged from 23.8 to 25.6 out of 26 for all subjects, EQIP scores were significantly lower for both ‘nutrition and dysphagia and children’ and ‘herbs and common cold’ than for ‘Mediterranean diet’ and ‘sports nutrition’ (scores not reported numerically, *p* < 0.001). DISCERN score was not significantly different between topics.	Not assessed
Shahar et al.^28^	Cancer	10.3% of the websites included all 10 main recommendations, while 28.9% included 5 of the 10. The content was found incomplete for 91% of the websites, producing poor accuracy scores.	*FRES* was 50%–59% (‘quite difficult’) for 44% of websites.
Herth et al.^29^	Cancer	Content scores ranged from 16.2% in self‐help group websites to 63.8% in online newspapers (lower scores depict higher quality).	Not assessed
Keaver et al.^18^	Cancer	The mean IPDAS score was 20.4/40.0.	*FRES* mean was 61.3 and *F‐KGLS* mean was 9.
Modave et al.^15^	Weight loss	The mean overall content score was 3.75/16. The mean score on nutrition content was 1.57 ± 0.99 out of 4.	Mean score of readability was 8.66 years.
		Less than 20% of websites included >50% accurate information on nutrition, physical activity or behavioural strategies for weight loss. Unsubstantiated claims were made by 54% of websites, particularly regarding nutritional information.	
Cardel et al.^50^	Weight loss	1.5% of websites scored 8/12 or higher, and 12% scored 6/12 or higher on content related to nutrition, behaviour change and physical activity. Unsubstantiated claims were made on 94% of websites; particularly regarding nutrition in 100% of the websites with unsubstantiated claims; regarding physical activity in 47%; and regarding behaviour change in 39%.	Not assessed
Guardiola‐Wanden‐Berghe et al.^51^	Weight loss and eating disorders	Mean quality scores for the websites on diet was 7.05 ± 0.15 out of 22 and for the websites on anorexia ⁄ bulimia, was 9.26 ± 0.16 out of 22. No websites were found that met the 22 quality criteria.	Not assessed
Meyer et al.^19^	Weight loss	None of the websites covered more than 10/18 (55.6%) of content criteria, 46.2% of websites covered >4/18 criteria (22.2%).	*FRES* scores ranged between 12 (hard to read) to 69 (easy to read).
		Among five covered themes (nutrition, physical activity, behaviour change, pharmacotherapy and surgery), nutrition was the best covered with a mean presence of 2/4 criteria. Only 25% of websites covered >2/4 criteria and only 1.5% of websites covered all nutrition criteria (4/4).	
		70.1% of websites recommended focusing on specific foods, 67.2% on avoiding specific foods and 47.8% covered energy balance, 9% advised on limiting salt intake.	
		Overall percentage of unsubstantiated claims was fairly high (63.4 ± 38.7). Nutrition and behavioural change were especially prone to unsubstantiated claims.	
Lambert et al.^46^	Renal disease	DISCERN score was poor (<2/5) for 49.6% of websites, fair (3/5) for 24.4% of websites and good (>4/5) for 26% of websites.	Median readability level of Grade 10 and median reader age 14 years old.
		**Content accuracy:** 73.2% of the total number of webpages evaluated, contained accurate information. For search term ‘diet for CKD’ 69.3% of websites provided accurate information. 87.5% of information on diet for PCKD was inaccurate.	
Traver et al.^39^	Kidney stones	Correct information was provided in 15% to 50% of sites.	Not assessed
Alfaro‐Cruz et al.^38^	Irritable bowel syndrome	For paediatric‐focused websites, the mean DISCERN score of information quality was 2.2/5.0 (low), while for adult‐focused websites it was 2.7/5.0 (low).	For paediatric‐focused websites mean score of readability was 2.8/5.0 (low), while for adult‐focused websites score was 3.4/5.0 (moderate).
			*F‐KGLS* was 11.0 ± 3.0 for paediatric‐focused websites and 11.4 ± 5.2, for adult‐focused websites, described as ‘difficult to read’.
Htet et al.^42^	Polycystic ovary syndrome (PCOS)	**Accuracy of information:** For dietary recommendations mean score was 23 ± 6 (out of 33); for physical activity mean score was 15 ± 5 (out of 27), for weight management mean score was 14 ± 3 (out of 24).	Not assessed
Beckett et al.^40^	Multiple sclerosis (MS)	**Quality of information:** Poorly backed scientific information for most webpages, often based on people's experience.	Not assessed
		**Caution on advice:** Most of the webpages disclaimed that the information provided did not constitute medical advice. 34.4% advised to consult a professional before making any changes to the diet.	
Ambra et al.^16^	COVID‐19	49.3% of webpages had a >50% content quality score (‘sufficient’), with homogenous distribution for higher and lower scorings.	39% of articles had a word count >1000, and 28% had a word count <500. 91% of articles had a Gulpease index <60 (‘difficult to read even for middle/secondary school education’).
Lee et al.^17^	Dementia	The total mean DISCERN was 50 ± 8 (good quality). DISCERN score was higher for sites with pages on treatment, or treatment and prevention than those on prevention (*p* = 0.001, *p* = 0.04, respectively).	Not assessed

Abbreviations: CKD, chronic kidney disease; COVID‐19, Coronavirus Disease 2019; EQIP, Ensuring Quality Information for Patients; F‐KGLS, Flesch‐Kincaid Grade Level Score; FRES, Flesch Reading Ease Score; IPDAS, International Patient Decision Aids Standards; PCDK, polycystic kidney disease; SMOG, Simple Measure of Gobbledygook.

Eight studies used DISCERN to assess information quality^17^, ^29^, ^31^, ^36^, ^38^, ^41^, ^46^, ^49^ and one of them used it in combination with another tool (EQIP).^31^ Two studies did not present results for the overall quality of all websites assessed.^17^, ^29^ On two occasions with topics of interest healthy diet for the general public and irritable bowel syndrome, the mean scores were low, rating the sites, overall, as poor.^36^, ^38^ In two other cases, 49.6% and 46.2% of websites providing dietary advice on diets for renal diseases and vegan diets, accordingly, were found to contain poor‐quality information with extensive or serious shortcomings.^46^, ^49^

### Results on accuracy based on websites' classification

Of the 29 studies, 24 classified websites and 21 reported results on the quality of the different website categories. In general, commercial sites were less accurate than non‐profit sites, which was attributed to the lack of scientific references to support them.^17^, ^34^, ^45^ In addition, organisational sites have been found to address more guidelines than government and commercial.^17^ Similarly, results were reported using the DISCERN tool showing that institutional websites and encyclopaedias scored higher than commercial, personal or newspaper pages,^31^, ^49^ and no differences were found between the sites when commercial pages were restricted to online health information providers or medical institutions/clinics.^17^ In one case where the DISCERN tool was used, significantly higher scores for support sites were found, followed by institutional and non‐pharmaceutical commercial ones.^36^ In congruence with the above, quantitative data showed that of all websites, non‐commercial ones had the highest proportion of articles with congruent advice.^15^, ^35^ In more detail, in three studies where the topic of interest was related to a clinical condition (i.e., coeliac disease, kidney stones, osteoporosis), medical sites were found to have correct information (51% of the sites) as opposed to 13%–16% of other sites, and 80% of websites owned by alternative health practitioners were found to contain inaccuracies.^30^, ^39^ In the above cases, further categorisation of institutional sites into organisational and educational ones showed that among all types of sites, the first scored the highest in terms of content quality.^41^ These findings have also been confirmed by a study assessing web‐based information for cancer patients.^68^ However, two studies with weight loss as a topic of interest, following the same methodology and classification groups, but differed in the language of content publication, found contradictory results.^15^, ^50^ The lowest content sub‐scores were reported for blogs in the study evaluating Spanish websites, while the opposite occurred for English‐speaking websites. Finally, on four occasions, the type of website was not correlated with the level of quality,^28^, ^32^, ^42^, ^51^ and it was shown that irrespective of type, sites that included authorship and affiliation delivered more accurate information than the rest.^51^

### Results on readability

Thirteen studies assessed the readability of websites' content using various tools and methods (Table 5). Six studies used the F‐KGL to assess readability and the levels ranged from 6.29 to 12.1 out of 18.^18^, ^34^, ^36^, ^38^, ^47^, ^49^ In two of them, assessing information on vegan and Mediterranean diet, the level was found to be 6.6 ± 1.7 and 7.2, indicating that the published material was within the recommended range of readability.^36^, ^49^ A higher score of 9–12.1, which is considered difficult to read, was found in three cases with topics of interest irritable bowel syndrome, cancer prevention or a healthy diet.^18^, ^34^, ^38^ Another study found mixed results on readability, with the term ‘medical nutrition therapy for diabetes’ retrieving sites of F‐KGL 11.5, and the terms ‘how to eat with diabetes’ and ‘diabetes diet’, 6.43 and 6.29, respectively.^47^ Of all the above studies, only two used solely this tool,^38^, ^47^ while the rest used it in combination with FRES.

Eight studies used FRES to assess readability.^18^, ^19^, ^28^, ^34^, ^36^, ^43^, ^45^, ^49^ Except for two studies,^18^, ^49^ which found a mean score of 63.3 ± 9.6 and 61.3/100, which is among the recommended, the rest showed that the content of the sites was quite difficult to read (50–59 FRES). Used solely,^28^, ^45^ or in combination with other tools (i.e., FOG, SMOG, F‐KGL), the same results on readability are produced.^34^, ^43^, ^49^ Two studies were an exception to that, in which F‐KGL and FRES showed different levels of readability.^18^, ^36^ Therefore, most of the FRE scores were interpreted as difficult to read, irrespective of the topic of interest, even though all were aimed at the general population (i.e., pregnancy, vegan diet). Wherever assessed, 0%–13.4% of sites had an easy‐to‐read FRE score (>60)^34^, ^43^, ^49^ and 33%–37.5% had the recommended 60–70.^36^, ^45^ These results are in agreement with those presented by the last three studies that used other indices or tools for this evaluation and having a medical nutrition therapy topic (i.e., weight loss, renal disease).^15^, ^16^, ^46^

## DISCUSSION

In total, 29 studies assessed the accuracy of dietary advice published on the web and regardless of the method used or the topic of interest, results showed that inconsistent or poor‐quality information is found on 16%–49.6% of websites, with this percentage rising to 54%–94% in the case of ‘weight loss’ information. Similar results were presented by Denniss et al. in their systematic review, who found that 47.7% of studies evaluating nutritional information accuracy published on websites, were classified as poor, and higher proportions of poor classifications for accuracy were observed for studies evaluating weight‐loss information (*n* = 4, 100%). Regarding the quality of information found in various types of websites, even though discrepancies were present, commercial sites were found to be inferior in terms of quality to the rest, while institutional and encyclopaedias scored the highest in our review. Similar to our results, discrepancies were also evident in the systematic review by Denniss et al.^20^ In that work, findings from the 38 studies evaluating content accuracy, showed that organisations and/or academic institutions provided the most accurate information in five studies, Wikipedia provided moderate information, and commercial websites' and blog's accuracy was poorer than that in other websites in four and five studies, respectively. Regarding our findings on blogs accuracy, a study evaluating Spanish websites found the lowest content accuracy for blogs and the opposite was presented in a study assessing English‐speaking websites. On the other hand, purely congruent with guidelines information was found in 16%–39.7% of websites in our study, while results from Denniss et al.^20^ systematic review showed that 13.2% of studies assessing accuracy found it to be on a good level.

Looking at the methodologies used by the studies included in the present systematic review, in most cases, researchers have decided on the keywords and used mainly the most popular browser and search engine (i.e., Google) to perform the search. On the other hand, there was a lot of variation regarding the topic investigated, the inclusion and exclusion criteria for website selection, the number of sites finally evaluated, the method of assessment, the presentation of the results, as well as the number and type of categories the sites were grouped. However, two main methods were used for content quality assessment: a self‐made scale to compare and score the accuracy of nutritional information published on sites to guidelines^15^, ^16^, ^17^, ^28^, ^29^, ^30^, ^32^, ^33^, ^34^, ^35^, ^36^, ^37^, ^39^, ^42^, ^43^, ^44^, ^45^, ^47^, ^48^, ^50^ and metadata encompassed in a validated tool (i.e., DISCERN).

Thirteen studies^15^, ^16^, ^18^, ^19^, ^28^, ^34^, ^36^, ^38^, ^43^, ^45^, ^46^, ^47^, ^49^ assessed the readability level, which, irrespective of the assessment tool used, was found, in most cases, higher than the recommended, meaning that most of the websites' content is difficult to read by the public. This is the first study that accumulates relevant data regarding nutrition information. Nevertheless, results from two systematic reviews on the readability level of online health or mental health information are in agreement with ours.^24^, ^69^ In both those reviews, it was found that the vocabulary and the sentence structure used were, in most cases, too complex for the general public, making the content hard to understand. Also, similar to our results, findings on readability reported in the systematic review by Boutemen and Miller showed that outcomes from FRES fell within a limited range of scores, while those from F‐KGL varied greatly.^18^, ^36^, ^49^, ^69^ Trying to explain the great variability in the results of F‐KGL it was shown, in our review, that for topics of interest for healthy populations (vegan or Mediterranean diet), the content was easier to comprehend, and the level of readability was between the recommended ranges,^36^, ^49^ with the exception of one such study, where the level of readability was higher than the proposed indicating difficult‐to‐read texts.^34^ On the other hand, when the content had to do with a medical condition (e.g., irritable bowel syndrome) or the keywords used for the search included a term (e.g., medical nutrition therapy), readability was rated with F‐KGL as difficult.^18^, ^38^

Accumulating evidence on the readability of dietary advice published on websites was one of the strengths of the present review as, to our knowledge, this is the first time that this has been done. Readability is considered a quality aspect by some researchers^70^ while it is also important to highlight the need for easy‐to‐understand information published online to avoid confusion. This review includes all research papers assessing the content accuracy of dietary advice published online, and it is the first one to focus solely on the written content of websites (instead of videos, or other social media). However, data from an already published systematic review, including also studies assessing information published on social media, found a similar proportion of poor, good or moderate content between websites and social media.^20^ Last, this review provides insight into the methodologies of studies evaluating nutritional information on the web, supporting, thus, future researchers willing to conduct similar research. However, some limitations are also present. First, the results of the included studies were presented as they were published in each study which means that no uniform way was employed by our team. This is in contrast to what has been previously done by Denniss et al.^20^ in their systematic review, who used a previously established coding framework to interpret the results of the included studies. Another limitation is the fact that content published on social media or videos was not assessed, and these are means of nutrition communication gaining publicity.^71^

Given the above, the dietary advice provided through other communication modalities such as social media or means such as videos needs to be assessed. At the same time, the lack of data regarding the quality of nutritional information on sites published in languages other than English highlights the importance of assessing information published in other languages. In the present review, 29 studies were retrieved evaluating the content quality of dietary advice on 18 different nutrition topics. This depicts the need for more primary research into the field to accumulate evidence of whether there are any differences in terms of content quality based on the dietary topic discussed. Last, the fact that our search excluded studies focusing on other aspects of dietary advice, such as taking supplements, may have impacted our results. However, our aim was to assess the dietary advice provided in terms of diet, which led to this exclusion, whereas a previously published systematic review that included all kinds of nutritional information published on the web found overall similar results to ours, showing that accuracy was low in almost half of the websites.^20^

## CONCLUSIONS

In accordance with previous evidence, inaccurate or mixed nutrition dietary advice was found on the web, regardless of the topic of interest, whereas the inaccuracy level was higher regarding weight loss, based on the results of this review. Looking at the content accuracy of websites classified into different groups, our results highlight the inferiority of information found on commercial sites over the rest in terms of agreement with the official guidelines, even though serious discrepancies are evident in the website classification among studies. All the above puts nutrition information seekers into a challenging position as it is more possible for a person who looks for dietary advice on the web to find inaccurate information. From a health professional's perspective, the results of the present review can be used as evidence‐based data on the percentage of accurate dietary advice found on the internet. Also, can scientifically support the advice provided by health professionals to the public to avoid commercial websites for this kind of information and use organisational or institutional ones.

Regarding readability, the information published seems inappropriate for people seeking dietary advice on the web, even though differences do exist between the tools used to assess it. As the content seems problematic, adding poor comprehension does not support nutrition literacy, a key factor in predicting dietary adherence.^72^ However, some optimism is warranted since from the various sources of nutritional information (websites, books, etc.) people seem to place the highest level of trust in nutrition scientists, professionals and scientific journals.^73^ So, to support dietary knowledge, publishing evidence‐based nutritional information with high readability may be an efficient way to provide accurate information to lay audiences, increase their trust, and thus support them in improving their dietary adherence.

## AUTHOR CONTRIBUTIONS

Both authors designed and conducted the systematic search for articles. Mary Micheli wrote the methods section, and tables and was responsible for formatting the manuscript. Evaggelia Fappa wrote the introduction, results, and discussion section. Both authors reviewed and edited the final manuscript.

## CONFLICT OF INTEREST STATEMENT

The authors declare no conflict of interest.

### TRANSPARENT PEER REVIEW

The peer review history for this article is available at https://www.webofscience.com/api/gateway/wos/peer-review/10.1111/jhn.13395.

## Data Availability

The data that support the findings of this study are available on request from the corresponding author. The data are not publicly available due to privacy or ethical restrictions.

## References

[1] European Commission. 53% of EU citizens sought health information online. 2020 [cited 2023 February 26]. https://ec.europa.eu/eurostat/web/products-eurostat-news/-/ddn-20200327-1

[2] Maon SN , Hassan NM , Seman SAA . Online health information seeking behavior pattern. Adv Sci Lett. 2017;23(11):10582–10585.

[3] Obasola OI , Agunbiade OM . Online health information seeking pattern among undergraduates in a Nigerian University. SAGE Open. 2016;6:2158244016635255.

[4] Pollard CM , Pulker CE , Meng X , Kerr DA , Scott JA . Who uses the internet as a source of nutrition and dietary information? An Australian population perspective. J Med Internet Res. 2015;17(8):e4548. 10.2196/jmir.4548 PMC4642382 26310192

[5] Fassier P , Chhim AS , Andreeva VA , Hercberg S , Latino‐Martel P , Pouchieu C , et al. Seeking health‐ and nutrition‐related information on the Internet in a large population of French adults: results of the NutriNet‐Santé study. Br J Nutr. 2016;115(11):2039–2046. 27081008 10.1017/S0007114516001355

[6] Bujnowska‐Fedak MM , Węgierek P . The impact of online health information on patient health behaviours and making decisions concerning health. Int J Environ Res Public Health. 2020;17(3):880. 32023828 10.3390/ijerph17030880 PMC7037991

[7] Asakura K , Todoriki H , Sasaki S . Relationship between nutrition knowledge and dietary intake among primary school children in Japan: combined effect of children's and their guardians' knowledge. J Epidemiol. 2017;27(10):483–491. 28576447 10.1016/j.je.2016.09.014 PMC5602805

[8] Vijaykumar S , McNeill A , Simpson J . Associations between conflicting nutrition information, nutrition confusion and backlash among consumers in the UK. Public Health Nutr. 2021;24(5):914–923. 33431107 10.1017/S1368980021000124 PMC10195575

[9] Sleeman KE , de Brito M , Etkind S , Nkhoma K , Guo P , Higginson IJ , et al. The escalating global burden of serious health‐related suffering: projections to 2060 by world regions, age groups, and health conditions. Lancet Glob Health. 2019;7(7):e883–e892. 31129125 10.1016/S2214-109X(19)30172-X PMC6560023

[10] Afshin A , Sur PJ , Fay KA , Cornaby L , Ferrara G , Salama JS , et al. Health effects of dietary risks in 195 countries, 1990–2017: a systematic analysis for the Global Burden of Disease Study 2017. Lancet. 2019;393(10184):1958–1972. 30954305 10.1016/S0140-6736(19)30041-8 PMC6899507

[11] Bujnowska‐Fedak MM , Waligóra J , Mastalerz‐Migas A . The internet as a source of health information and services. Adv Exp Med Biol. 2019;1211:1–16. 31273574 10.1007/5584_2019_396

[12] Daraz L , Morrow AS , Ponce OJ , Beuschel B , Farah MH , Katabi A , et al. Can patients trust online health information? A meta‐narrative systematic review addressing the quality of health information on the internet. J Gen Intern Med. 2019;34(9):1884–1891. 31228051 10.1007/s11606-019-05109-0 PMC6712138

[13] Jia X , Pang Y , Liu LS . Online health information seeking behavior: a systematic review. Healthcare. 2021;9(12):1740. 34946466 10.3390/healthcare9121740 PMC8701665

[14] Thapa DK , Visentin DC , Kornhaber R , West S , Cleary M . The influence of online health information on health decisions: a systematic review. Patient Educ Couns. 2021;104(4):770–784. 33358253 10.1016/j.pec.2020.11.016

[15] Modave F , Shokar NK , Peñaranda E , Nguyen N . Analysis of the accuracy of weight loss information search engine results on the internet. Am J Public Health. 2014;104(10):1971–1978. 25122030 10.2105/AJPH.2014.302070 PMC4167115

[16] Ambra R , Canali R , Pastore G , Natella F . Covid‐19 and diet: an evaluation of information available on internet in Italy. Acta Biomed. 2021;92(1):e2021077. 33682806 10.23750/abm.v92i1.11033 PMC7975965

[17] Lee J , Nguyen J , O'Leary F . Content, quality and accuracy of online nutrition resources for the prevention and treatment of dementia: a review of online content. Dietetics. 2022;1(3):148–163.

[18] Keaver L , Huggins MD , Chonaill DN , O'Callaghan N . Online nutrition information for cancer survivors. J Hum Nutr Diet. 2023;36:415–433. 36177612 10.1111/jhn.13095

[19] Meyer S , Elsweiler D , Ludwig B . Assessing the quality of weight loss information on the German language web. Mov Nutr Heal Dis. 2020;4:39–52. 10.5283/MNHD.26

[20] Denniss E , Lindberg R , McNaughton SA . Quality and accuracy of online nutrition‐related information: a systematic review of content analysis studies. Public Health Nutr. 2023;26(7):1345–1357. 37138366 10.1017/S1368980023000873 PMC10346027

[21] Anderson HL , Moore JE , Millar BC . Comparison of innovative communication approaches in nutrition to promote and improve health literacy. Ulster Med J. 2022;91(2):85–91. 35722219 PMC9200103

[22] Murakami K , Shinozaki N , Okuhara T , McCaffrey TA , Livingstone MBE . Prevalence and correlates of dietary and nutrition information seeking through various web‐based and offline media sources among Japanese adults: web‐based cross‐sectional study. JMIR Public Health Surveill. 2024;10(1):e54805. 38354021 10.2196/54805 PMC10902774

[23] Adamski M , Truby H , M. Klassen K , Cowan S , Gibson S . Using the internet: nutrition information‐seeking behaviours of lay people enrolled in a massive online nutrition course. Nutrients. 2020;12(3):750. 32178291 10.3390/nu12030750 PMC7146568

[24] Daraz L , Morrow AS , Ponce OJ , Farah W , Katabi A , Majzoub A , et al. Readability of online health information: a meta‐narrative systematic review. Am J Med Qual. 2018;33(5):487–492. 29345143 10.1177/1062860617751639

[25] Page MJ , McKenzie JE , Bossuyt PM , Boutron I , Hoffmann TC , Mulrow CD , et al. The PRISMA 2020 statement: an updated guideline for reporting systematic reviews. Syst Rev. 2021;10(1):89. 33781348 10.1186/s13643-021-01626-4 PMC8008539

[26] Cooke A , Smith D , Booth A . Beyond PICO: the SPIDER tool for qualitative evidence synthesis. Qual Health Res. 2012;22(10):1435–1443. 22829486 10.1177/1049732312452938

[27] Methley AM , Campbell S , Chew‐Graham C , McNally R , Cheraghi‐Sohi S . PICO, PICOS and SPIDER: a comparison study of specificity and sensitivity in three search tools for qualitative systematic reviews. BMC Health Serv Res. 2014;14(1):579. 25413154 10.1186/s12913-014-0579-0 PMC4310146

[28] Shahar S , Shirley N , Noah SA . Quality and accuracy assessment of nutrition information on the Web for cancer prevention. Inform Health Soc Care. 2013;38(1):15–26. 22957981 10.3109/17538157.2012.710684

[29] Herth N , Kuenzel U , Liebl P , Keinki C , Zell J , Huebner J . Internet information for patients on cancer diets – an analysis of German websites. Oncol Res Treat. 2016;39(5):273–281. 27173518 10.1159/000445861

[30] England CY , Nicholls AM . Advice available on the Internet for people with coeliac disease: an evaluation of the quality of websites. J Hum Nutr Diet. 2004;17(6):547–559. 15546433 10.1111/j.1365-277X.2004.00561.x

[31] Gkouskou K , Markaki A , Vasilaki M , Roidis A , Vlastos I . Quality of nutritional information on the Internet in health and disease. Hippokratia. 2011;15(4):304–307. 24391409 PMC3876843

[32] Gholizadeh Z , Papi A , Ashrafi‐rizi H , Shahrzadi L , Hasanzadeh A . Quality evaluation of Persian nutrition and diet therapy websites. J Educ Health Promot. 2017;6(1):48. 28616415 10.4103/jehp.jehp_83_14 PMC5470293

[33] Davison K . The quality of dietary information on the World Wide Web. Clin Perform Qual Health Care. 1997;5(2):64–66. 10167213

[34] Sutherland LA , Wildemuth B , Campbell MK , Haines PS . Unraveling the web: an evaluation of the content quality, usability, and readability of nutrition web sites. J Nutr Educ Behav. 2005;37:300–305. 16242061 10.1016/s1499-4046(06)60160-7

[35] Ostry A , Young ML , Hughes M . The quality of nutritional information available on popular websites: a content analysis. Health Educ Res. 2007;23(4):648–655. 17897928 10.1093/her/cym050

[36] Hirasawa R , Saito K , Yachi Y , et al. Quality of internet information related to the Mediterranean diet. Public Helath Nutr. 2011;15(1):885–893. 10.1017/S1368980011002345 21923978

[37] Hirasawa R , Yachi Y , Yoshizawa S , Horikawa C , Heianza Y , Sugawara A , et al. Quality and accuracy of Internet information concerning a healthy diet. Int J Food Sci Nutr. 2013;64(8):1007–1013. 23863089 10.3109/09637486.2013.812620

[38] Alfaro‐Cruz L , Kaul I , Zhang Y , Shulman RJ , Chumpitazi BP . Assessment of quality and readability of internet dietary information on irritable bowel syndrome. Clin Gastroenterol Hepatol. 2019;17(3):566–567. 29800724 10.1016/j.cgh.2018.05.018 PMC6250590

[39] Traver MA , Passman CM , Leroy T , Passmore L , Assimos DG . Is the internet a reliable source for dietary recommendations for stone formers. J Endourol. 2009;23(4):715–717. 19335145 10.1089/end.2008.0490 PMC2827241

[40] Beckett JM , Bird M‐L , Pittaway JK , Ahuja KD . Diet and multiple sclerosis: scoping review of web‐based recommendations. Interact J Med Res. 2019;8(1):e10050. 30626570 10.2196/10050 PMC6329429

[41] Joshi A , Bhangoo RS , Kumar K . Quality of nutrition related information on the internet for osteoporosis patients: a critical review. Technol Health Care. 2011;19(6):391–400. 22129940 10.3233/THC-2011-0643

[42] Htet T , Cassar S , Boyle J , Kuczynska‐Burggraf M , Gibson‐Helm M , Chiu WL , et al. Informing translation: the accuracy of information on websites for lifestyle management of polycystic ovary syndrome. Semin Reprod Med. 2018;36(1):80–85. 30189455 10.1055/s-0038-1667309

[43] Storr T , Maher J , Swanepoel E . Online nutrition information for pregnant women: a content analysis. Matern Child Nutr. 2017;13(2):e12315. 27353248 10.1111/mcn.12315 PMC6865949

[44] Cannon S , Lastella M , Vincze L , Vandelanotte C , Hayman M . A review of pregnancy information on nutrition, physical activity and sleep websites. Women Birth. 2020;33(1):35–40. 30905558 10.1016/j.wombi.2018.12.007

[45] Sidnell A , Nestel P . UK Internet antenatal dietary advice: a content accuracy and readability analysis. Br J Nutr. 2020;124:1061–1068. 32536348 10.1017/S0007114520002135

[46] Lambert K , Mullan J , Mansfield K , Koukomous A , Mesiti L . Evaluation of the quality and health literacy demand of online renal diet information. J Hum Nutr Diet. 2017;30(5):634–645. 28211108 10.1111/jhn.12466

[47] Bernard S , Cooke T , Cole T , Hachani L , Bernard J . Quality and readability of online information about type 2 diabetes and nutrition. JAAPA. 2018;31(11):41–44. 10.1097/01.JAA.0000546481.02560.4e 30358679

[48] Post RE , Mainous III, AG . The accuracy of nutrition information on the internet for type 2 diabetes. Arch Intern Med. 2010;170(16):1504–1505. 20837841 10.1001/archinternmed.2010.289

[49] El Jassar OG , El Jassar IN , Kritsotakis EI . Assessment of quality of information available over the internet about vegan diet. Nutr Food Sci. 2019;49(6):1142–1152.

[50] Cardel MI , Chavez S , Bian J , Peñaranda E , Miller DR , Huo T , et al. Accuracy of weight loss information in Spanish search engine results on the internet. Obesity. 2016;24(11):2422–2434. 27653438 10.1002/oby.21646 PMC5117437

[51] Guardiola‐Wanden‐Berghe R , Gil‐Pérez JD , Sanz‐Valero J , Wanden‐Berghe C . Evaluating the quality of websites relating to diet and eating disorders. Health Inf Libr J. 2011;28(4):294–301. 10.1111/j.1471-1842.2011.00961.x 22051128

[52] National Institutes of Health, National Heart Lung and Blood Institute. *Your guide to lowering your blood pressure with DASH*. 2006 [cited 2011 June 30]. http://www.nhlbi.nih.gov/health/public/heart/hbp/dash/new_dash.pdf

[53] U.S. Department of Health and Human Services, U.S. Department of Agriculture. *Dietary guidelines for Americans 2005*. 2005.

[54] Center for Nutrition Policy and Promotion. *Dietary guidelines for Americans 2010*. 2010.

[55] U.S. Department of Health and Human Services, U.S. Department of Agriculture. *2015–2020 Dietary Guidelines for Americans*. 8th ed. 2015.

[56] Powers MA , Bardsley J , Cypress M , Duker P , Funnell MM , Hess Fischl A , et al. Diabetes self‐management education and support in type 2 diabetes: a joint position statement of the American Diabetes Association, the American Association of Diabetes Educators, and the Academy of Nutrition and Dietetics. Diabetes Care. 2015;38(7):1372–1382. 26048904 10.2337/dc15-0730

[57] Charnock D , Shepperd S , Needham G , Gann R . DISCERN: an instrument for judging the quality of written consumer health information on treatment choices. J Epidemiol Community Health. 1999;53:105–111. 10396471 10.1136/jech.53.2.105 PMC1756830

[58] Moult B , Franck LS , Brady H . Ensuring Quality Information for Patients: development and preliminary validation of a new instrument to improve the quality of written health care information. Health Expect. 2004;7(2):165–175. 15117391 10.1111/j.1369-7625.2004.00273.x PMC5060233

[59] Kincaid PJ , Fishburne RPJ , Rogers RL , Chissom BS . Derivation of new readability formulas (automated readability index, fog count and flesch reading ease formula) for navy enlisted personnel. Millington (Tennessee): Institute for Simulation and Training, University of Central Florida; 1975. http://www.dtic.mil/dtic/tr/fulltext/u2/%0Aa006655.pdf%0A

[60] National Institutes of Health. How to write easy to read health materials. 2017 [cited 2018 February 28]. http://www.nlm.nih.gov/medlineplus/etr.html

[61] Flesch R . A new readability yardstick. J Appl Psychol. 1948;32(3):221–233. 18867058 10.1037/h0057532

[62] Gunning R . The technique of clear writing. New York: McGraw‐Hill; 1952.

[63] Mc Laughlin, HG SMOG grading—a new readability formula. J Read. 1969;12(8):639–646.

[64] Coleman M , Liau TL . A computer readability formula designed for machine scoring. J Appl Psychol. 1975;60(2):283–284.

[65] Smith EA , Senter RJ . Automated readability index. Cincinnati: Wright‐Patterson Air Force Base; 1967.

[66] O'hayre J , Gobbledygook has gotta go. U.S. Government Printing Office; 1966 [cited 2023 February 27]. https://books.google.com/books?hl=en&lr=&id=1yTeNg9bxGUC&oi=fnd&pg=PA4&dq=Gobbledygook+Has+Gotta+Go&ots=VRjXHDexmW&sig=yLB6zmIU7LeQDyuDrz5CmUtCuks

[67] Lucisano P , Piemontese ME . GULPEASE: Una formula per la predizione della difficoltà dei testi in lingua italiana. Sc e città. 1988;3:110–124.

[68] Llaha F , Ribalta A , Arribas L , Bellver M , Roura E , Guillén‐Rey N , et al. A review of web‐based nutrition information in Spanish for cancer patients and survivors. Nutrients. 2022;14(7):1441. 35406054 10.3390/nu14071441 PMC9003392

[69] Boutemen L , Miller AN . Readability of publicly available mental health information: a systematic review. Patient Educ Couns. 2023;111:107682. 36944285 10.1016/j.pec.2023.107682

[70] Zhang Y , Sun Y , Xie B . Quality of health information for consumers on the web: a systematic review of indicators, criteria, tools, and evaluation results. J Assoc Inf Sci Technol. 2015;66(10):2071–2084.

[71] Doub AE , Small ML , Levin A , LeVangie K , Brick TR . Identifying users of traditional and Internet‐based resources for meal ideas: an association rule learning approach. Appetite. 2016;103:128–136. 27067739 10.1016/j.appet.2016.04.006

[72] Taylor MK , Sullivan DK , Ellerbeck EF , Gajewski BJ , Gibbs HD . Nutrition literacy predicts adherence to healthy/unhealthy diet patterns in adults with a nutrition‐related chronic condition. Public Health Nutr. 2019;22(12):2157–2169. 31146797 10.1017/S1368980019001289 PMC6827561

[73] Ruani MA , Reiss MJ , Kalea AZ . Diet‐nutrition information seeking, source trustworthiness, and eating behavior changes: an international web‐based survey. Nutrients. 2023;15(21):4515. 37960169 10.3390/nu15214515 PMC10649819

